# The digestion time for salmon louse (*Lepeoptheirus salmonis*) in lumpfish (*Cyclopterus lumpus)* in relation to freshness, developmental stage, and temperature

**DOI:** 10.1371/journal.pone.0311073

**Published:** 2025-03-13

**Authors:** Kirstin Eliasen, Sandra Ljósá Østerø, Tróndur Tummasarson Johannesen, Esbern Jóannes Patursson, Ása Jacobsen, Agnes Mols Mortensen, Marner Nolsøe, Ása Johannesen

**Affiliations:** 1 Department of Fish Health, Firum, Hvalvík, Faroe Islands; 2 Department of Fjord Dynamics, Firum, Hvalvík, Faroe Islands; 3 Hiddenfjord, Sandavágur, Faroe Islands; 4 Department of Biotechnology, Firum, Hvalvík, Faroe Islands; 5 TARI – Faroe Seaweed, 100 Tórshavn, Faroe Islands; 6 Bakkafrost, Glyrar, Faroe Islands; 7 Department of Ecology, Firum, Hvalvík, Faroe Islands; Universitat Politècnica de València, SPAIN

## Abstract

Sea lice infestations cause significant economic losses in the Atlantic salmon aquaculture industry. To biologically control sea lice at farming sites, cleaner fish such as lumpfish are employed. However, the efficacy of lumpfish is under constant debate, primarily due to limited knowledge of digestion times, which makes it difficult to interpret the number of salmon lice found in the stomach contents of dissected lumpfish.

The aim of this study was to provide quantitative estimates of the degradation of salmon lice over a period of 12 days. After an acclimation period of approximately one week, batches of eight lumpfish (average weight 94.3 g, SD ± 33.2) were fed salmon lice and arranged in tanks. Each batch received six large mobile lice and two adult female lice. Samplings were conducted at 24-hour intervals during the first four days and at 48-hour intervals over the remaining eight days. The experiment was conducted twice, each at a different temperature regime (6°C and 9°C), using live lice in both trials. To investigate if the freshness of the louse influenced degradation and digestion, the setup was replicated in the 9°C experiment with lice that had been stored frozen at -80°C, with an additional 12-hour sampling point for comprehensive observation.

The analysis of salmon lice revealed expected digestion times of 6.4 days and 12.9 days for large mobile and adult female salmon lice, respectively. Temperature and lice freshness did not seem to influence digestion times, but the developmental stage of the lice did. The findings of this study can be used to estimate the cleaning efficacy of lumpfish based on the stomach contents.

## Introduction

Salmon lice, *Lepeophtheirus salmonis,* have posed a serious problem for the Atlantic salmon farming industry since the 1970s [1], with their economic impact surpassing that of any other farmed salmon parasite [2]. The increasing resistance of sea lice to medical treatments necessitated the exploration of alternative, non-pharmaceutical methods [3–6], and as a result, the use of cleaner fish emerged as a method for controlling sea lice [7].

Several fish species have been identified as effective cleaners, particularly among the wrasses (labridae) [8–9]. However, the main wrasse species currently used for biological delousing (*Ctenolabrus rupestris*, *Symphodus melops*, and *Labrus bergylta)* are temperature-sensitive, making them unsuitable for use at low temperatures [10]. In contrast, lumpfish (*Cyclopterus lumpus*) have proven to be effective cleaners at lower temperatures [11–12]. Due to the relatively low temperatures and the absence of native wrasse species, lumpfish is the only cleaner fish used in the Faroe Islands [13].

Since the onset of using lumpfish as cleaner fish, their cleaning effort has shown to vary greatly, both spatially, seasonally and with size [11, 13–17]. According to Staven et al. [17], there are two primary approaches to assessing the cleaning efficacy of lumpfish. The first method involves indirectly estimating efficacy by comparing sea lice infestation levels in cages with and without lumpfish. This approach has been demonstrated in controlled experiments [12,18] and modelling studies [19]. However, both methods are significantly influenced by the complexity of sea lice population dynamics at both the site and on cage level [15]. The second, more direct method involves reviewing data and personal experiences from fish farmers, as conducted by Imsland and Reynolds [20].

An alternative approach to measuring cleaning efficacy is to investigate and count the presence of lice in the stomach contents of lumpfish, as has been done in numerous studies [11,13,15,16]. However, this method must be combined with assumptions about digestion time, underscoring the importance of understanding the digestion time for salmon lice in lumpfish.

Additionally, the use of cleaner fish for biological control has raised ethical considerations, particularly regarding the welfare and high mortality rates frequently observed when cleaner fish are stocked in sea cages [21–23]. Given these concerns about the efficacy and welfare of cleaner fish in commercial sea cages, forceful evidence is required to justify and guide their use in the industry.

Here, we investigate the digestion times of salmon lice in lumpfish stomachs primarily to assess the effects of temperature and louse life stage. Salmon lice were inserted into the stomachs of live lumpfish and left to incubate for predetermined time intervals. Following incubation, the lumpfish were dissected to determine the digestion status of the lice.

## Material and methods

### Ethical statement

The use of lumpfish for experimental purposes was accepted by the Faroese Food and Veterinary Authority (HFS 23/0331-5 and 23/00331-11). Additionally, Firum’s Animal Experimentation Ethics Committee reviewed and approved the study (approval number 2023_01). The committee’s decision was based on the anticipated welfare benefits for lumpfish in aquaculture and the minimal suffering projected to result from the study.

Lumpfish were sourced from commercial salmon farms and transferred to the experimental facilities in tanks with aerated seawater by car. All fish were housed in communal tanks, fed ad libitum throughout the study, and provided with shelters. Sedation was performed in dark buckets with aerated seawater using a low dose of MS-222 (100 mg/L, according to Skår et al. [24]) until the fish showed mild signs of sedation (poor balance) to minimize stress during the experimental procedure. Euthanasia was carried out with an overdose of MS-222 (1 g/L, according to Skår et al. [24]) for a minimum of 15 minutes, after which the fish were dissected immediately to ensure they did not regain consciousness.

Any fish showing signs of poor welfare, such as injuries or abnormal behaviour, would be euthanized before the end of the trial. However, all the fish in this study were healthy and did not warrant any intervention.

### Research animals

The lumpfish used in this study were collected from salmon cages at a Hiddenfjord farming site, one to two months post deployment. The fish originally came from Benchmark Genetics Iceland hf in Iceland and were F1 from wild caught broodstock. The lumpfish were transported to the Faroe Islands in marine shipping containers.

At arrival, the lumpfish (*n* = 208 split in two rounds of trials with 144 and 64 lumpfish each) were distributed among experimental housing tanks. The tanks were white-bottomed, black-sided fiberglass tanks with a capacity of 125 litres, measuring approximately 50 cm × 50 cm × 50 cm. Maximum biomass in each tank was 10 kg/m^3^. All tanks had a flow-through system with aerated seawater, maintaining a flow rate of two litres per minute, equating to a full tank exchange every hour. Each tank was equipped with shelters made of black PE drainage pipes cut in half lengthwise and hung vertically in pairs (40 cm long, with a total area of 0.12 m²). The overhead lights were set to a 12:12 light:dark schedule. The lumpfish were fed to satiation once or twice daily throughout both rounds of trials using the same commercial feed pellets provided at the farming site. The feed (Skretting, Clean Lumpfish 3, 3 mm) was high in protein and low in fat. To ensure that any salmon lice consumed at the farming site did not affect the study results, the lumpfish were not fed a salmon louse until seven to nine days after their arrival at the research facility.

Lumpfish were intentionally chosen by eye to represent a range in sizes from deployment size up to the size when anecdotally, they decrease their louse cleaning activity. By the end of the experiment, the lumpfish were weighed and had a mean weight of 94.3 g (Min 46 g, Max 268 g), with a standard deviation (SD) of 33.3 g.

Salmon lice used in the study were collected from salmon farms during the mandated sea lice counts, with the collection occurring within days before the start of the trial. The lice were sampled directly from the salmon, placed in buckets filled with seawater, and transported by car to the research facility. To ensure the lice remained alive until the experiment began, aeration was provided to the buckets, and the water was maintained at *in situ* temperatures. However, for comparative purposes, a fraction of the sampled salmon lice was frozen at -80°C immediately upon arrival at the research facility.

### Experimental setup and sampling

The study consists of three treatments (freshness, developmental stage and temperature) divided into two trials conducted at separate times. The first trial compared digestion time of frozen and live salmon lice where half the lumpfish were fed live lice, and the other half were fed frozen lice. Additionally, to compare digestion time of developmental stage of lice, one quarter of the lumpfish (two out of eight fish per sampling point) were fed an adult female salmon louse, whereas the rest were fed a large mobile salmon louse. Subsequently, the same trial was carried out at a lower temperature but using only live salmon lice. The decision to not use frozen lice in the second trial was made in order to minimise the use of animals, as the initial trial had clear enough results for frozen lice and establishing the effect of temperature on digestion of live lice had a higher priority.

To facilitate handling, lumpfish were anesthetized using a solution containing 100 mg/L MS-222 (Tjaldurs Apotek, Tórshavn, Faroe Islands) until they exhibited signs of sedation, such as cessation of swimming, loss of equilibrium, and lack of responsiveness, in accordance with the protocol outlined by Skår et al. [24]. Subsequently, and in batches of eight, the sedated lumpfish were administered a single salmon louse via oral insertion, guiding it past the oesophagus using rounded-tipped forceps. Within each batch, two lumpfish were administered an adult female salmon louse, while the remaining six were fed a large mobile salmon louse, i.e. a preadult II or adult male salmon lice (52 adult females and 156 large mobile).

After receiving the salmon lice, each batch of lumpfish (*n* = 8) was assigned to a designated tank (18 tanks in total), representing sampling points at intervals of 0.5, 1, 2, 3, 4, 6, 8, 10, or 12 days after the feeding event (*n* = 72 for frozen lice and live lice, respectively). All lumpfish were also offered their usual lumpfish feeds throughout the incubation period to ensure that digestion activity remained normal and like that at a farming site. The second trial did not include the 0.5-day sampling point, as all lice were recovered after 24 hours at the warmer temperature in the previous trial (*n* = 64, eight tanks). The selection of sampling points was based on a previous study, which found that 66% of adult male salmon lice could be visually identified three days after being consumed by lumpfish [25]. All fish recovered seamlessly from the sedation, displaying no signs of distress or adverse effects from the procedure, and there were no recorded mortalities throughout the experiment.

Temperature readings were recorded using RBR Solo^3^ D temperature loggers, capturing data at 10-minute intervals. The experiment was conducted twice, each at a different temperature regime: first, from December 9, 2023, to December 21, 2023, with an average temperature of 9.01°C (minimum: 7.64°C, maximum: 9.52°C) and with an average lumpfish weight of 89.7 g, with a standard deviation (SD) of 20.9 g; and second, from March 1, 2024, to March 13, 2024, with an average temperature of 6.25°C (minimum: 5.14°C, maximum: 6.68°C) and with an average lumpfish weight of 104.5 g, with a standard deviation (SD) of 49.7 g.

At each designated sampling point, following the protocol established by Skår et al. [24], lumpfish underwent euthanasia through a 15-minute exposure to a 1 g/L MS-222 solution. Subsequently, their stomachs were carefully dissected for content assessment. The measurements conducted included time elapsed since feeding, weight, length, and determination of sex.

**Fig 1:**
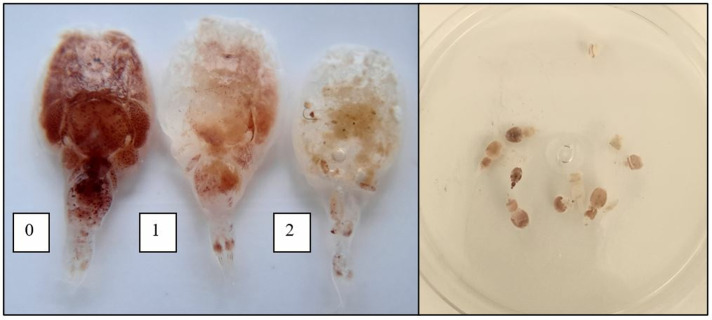
Degradation categories for salmon lice were defined as follows: (0) no apparent degradation, (1) slight signs of degradation, and (2) significant signs of degradation (left). The 11 adult female salmon lice and one mobile salmon louse found in a lumpfish stomach at four days incubation time (right).

Stomach contents were categorized based on the presence of (1) adult female salmon lice, (2) mobile stage of salmon lice, and (3) lumpfish feed. Furthermore, each salmon louse discovered was evaluated for signs of degradation, with categorizations of (0) no apparent degradation, (1) slight signs of degradation, and (2) significant signs of degradation. A rating of 1 indicated partial digestion of the soft tissue, while a rating of 2 signified complete digestion of the soft tissue (Fig 1). A rating of 3 signified the point at which the louse was no longer visually detectable.

Despite being housed at the research facility for at least a week before the experiment began, two lumpfish that had received a frozen louse were found to have ingested additional salmon lice. One lumpfish contained 11 adult female salmon lice and one large mobile salmon louse, while the other had one adult female salmon louse in its stomach at four and ten days of incubation, respectively. The lumpfish with a single adult female louse was identified because the number of adult females retrieved exceeded the number originally fed. Fortunately, all adult females sampled on day ten exhibited the same level of degradation, allowing a lumpfish to be reclassified as having been fed a large mobile salmon louse. In contrast, the lumpfish with numerous lice in its stomach was excluded from the data analysis (n = 207), as it was impossible to accurately determine the developmental stage or degradation category of the louse originally fed to it. While we cannot entirely rule out the possibility that the lumpfish with one adult female salmon louse was misfed, the significant number of lice found in the other fish strongly suggests that it had retained salmon lice ingested at the farming site for at least 11 days. If the fish with one adult female louse in its stomach was not the result of a misfeed, this lumpfish retained an adult female salmon louse for at least 17 days post-ingestion.

### Statistical analysis

Survival analysis was used for estimating the probability of recovering salmon lice over time. The response variable was binary (i.e., identified, or unidentified) at a given time, and the data were a mix of left- and right-censored data, indicating that the true identification times were unknown and could have occurred either before or after a sampling point. The analysis was done in two stages: A non-parametric survival analysis was done to provide an overview of survival probabilities using a Kaplan-Meier estimator. Afterwards, a parametric survival regression using the Weibull distribution was performed to model the survival probabilities over time. First, a combined survival regression model was fitted to assess the overall significance of developmental stage, temperature, and freshness as predictors. Secondly, the nonsignificant predictors were excluded from the model, and separate regressions for the two developmental stages was fitted. This approach allowed for different scale parameters for each developmental stage, which captured the characteristics of each stage better.

The analysis was performed using the survfit and survreg functions in the survival package in R (www.r-project.org) [26]. For estimation of mean digestion times, the survival curves were integrated. An alternative approach for analysing these data would be to carry out a logistic regression with a binary response variable (louse found or not) and interaction terms between time post-ingestion and the various fixed variables as predictors. This approach would be similar to that used by Staven et al. [17], and while not inappropriate, the authors chose to use survival analysis instead and the results were very similar. For the sake of comparison, the results from carrying out the analysis using logistic regression are presented as supplementary data (S1).

To determine factors influencing the degradation levels of salmon lice, a cumulative link model (CLM) analysis was used to investigate the interaction between time and freshness (frozen vs live lice), temperature and developmental stage on degradation level. The model included degradation level as ordinal response, and interaction terms between time and each of the factors. The CLM was fitted using the clm function from the ordinal package in R (www.r-project.org) [27].

In all statistical tests, results were considered significant at p < 0.05.

## Results

### Salmon louse recovery and degradation over time

**Fig 2:**
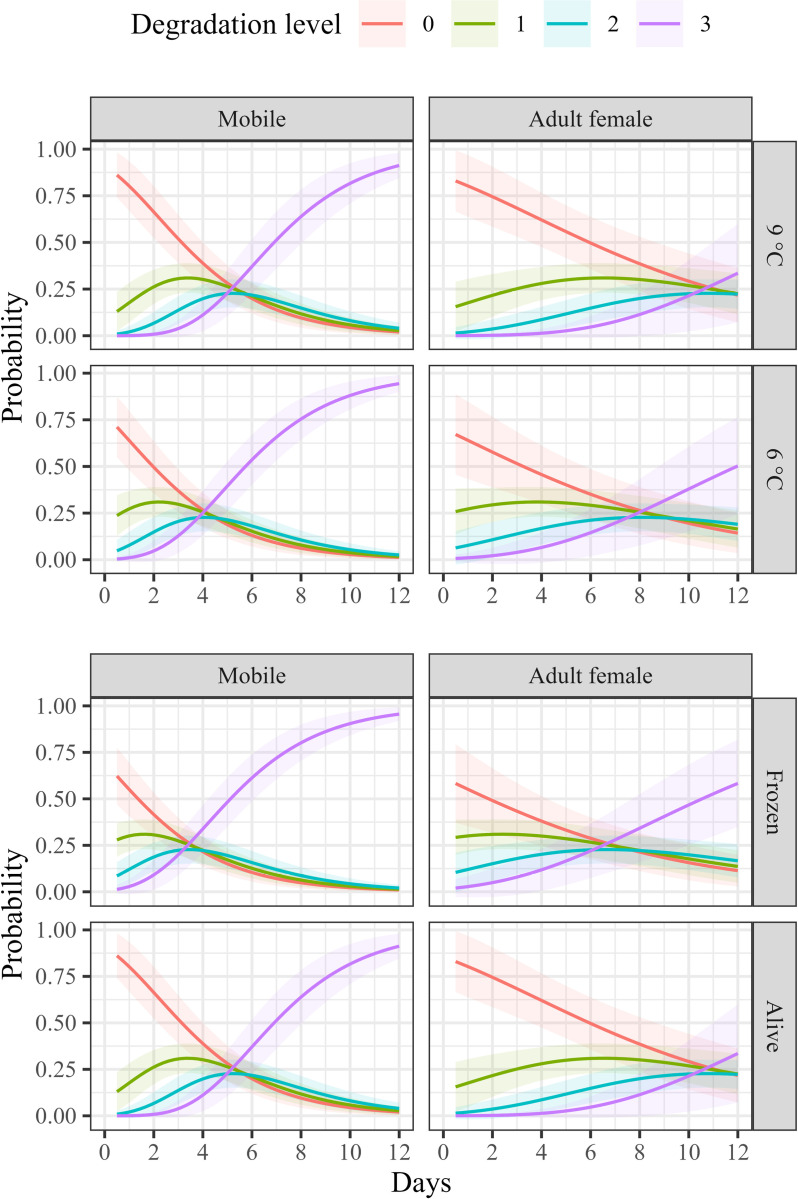
Probability of recovering lice at various degradation levels over time for the two developmental stages, presented in relation to temperature (upper panel) and freshness (lower panel). A degradation level of 3 indicates that the louse was no longer visually detectable.

Fig 2 illustrates the probability of recovering lice at various degradation levels over time for the two developmental stages in relation to temperature (upper panel) and freshness (lower panel). The data suggest that the degradation trend is primarily influenced by the developmental stage of the lice, with temperature and the freshness of the lice playing a relatively minor role.

### Factors influencing salmon louse recovery

**Fig 3:**
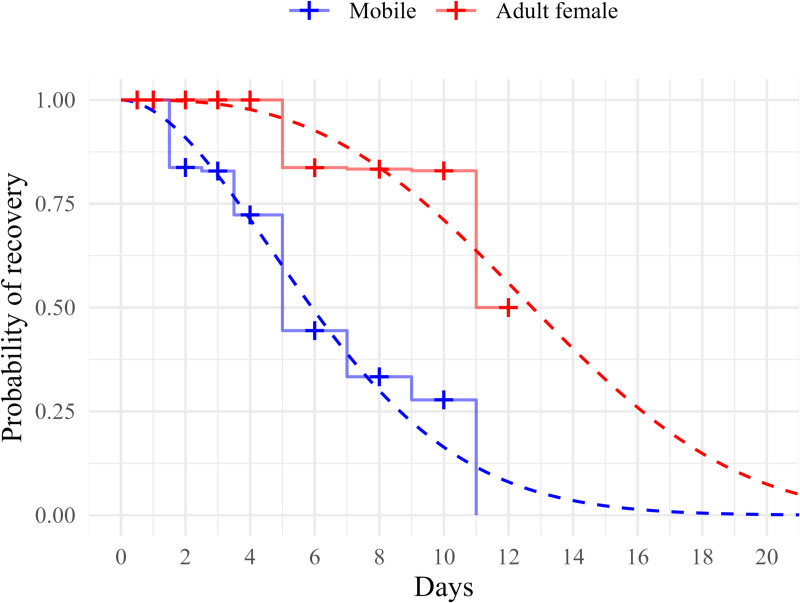
The probability of recovering adult female and mobile salmon lice from lumpfish stomachs across different sampling times. Full lines are from the non-parametric survival analysis, and broken lines are from the fitted parametric survival regression. ‘+’ indicates sampling points.

The survival regression analysis showed no significant difference in the recovery of sea lice between the two temperatures (deviance_1,202_ = 1.380, p = 0.240) or freshness (deviance_1,203_ = 3.122, p = 0.077). These p-values indicate that neither temperature nor freshness (frozen or alive) significantly influenced the recovery rates of sea lice. The analysis showed a significant difference (deviance_1,204_ = 25.669, p < 0.001) in the proportion of lice recovered between the two developmental stages of salmon lice. The probability of recovering a mobile salmon louse thus decreased significantly faster over time compared to adult female salmon lice (Fig 3).

All lice were recovered within the first day. However, from the second day onward, the proportion of mobile salmon lice steadily decreased, and by day 12, no mobile lice were recovered. In contrast, all adult females were recovered until day four, and by day 12, half of the adult females were recovered.

### Expected digestion times

Integrating the survival curves resulted in the expected digestion times of 6.4 days for large mobile salmon lice and 12.9 days for adult female salmon lice.

### Factors influencing salmon louse degradation

The interaction between time and developmental stage on degradation level was significant (z = -3.540, n = 207, p = <0.001), indicating that mobile lice degraded faster than adult female lice. No other significant interactions were found, indicating that temperature (z = -1.003, n = 207, p = 0.316) and freshness (z = 0.493, n = 207, p = 0.622) did not affect degradation level over time. However, there was a significant main effect of temperature (z = 2.088, n = 207, p = 0.037), indicating more rapid degradation at the start of digestion, and freshness (z = -3.242, n = 207, p = 0.001), indicating that frozen lice start out more degraded than live lice.

## Discussion

Understanding the digestion time of salmon lice in lumpfish is crucial for assessing their cleaning efficacy. This knowledge enables the estimation of number of salmon lice consumed per unit of time based on the number of salmon lice found in stomach contents [15]. Given that the welfare of lumpfish is frequently compromised in sea cages [21–23], obtaining accurate information on the digestion time of salmon lice in lumpfish is urgently needed. This study provides essential insights, offering robust guidance for further use of lumpfish in the aquaculture industry.

To the authors’ knowledge, this is the first study to investigate the probability of recovering salmon lice fed to the lumpfish alive as a function of time since feeding. While Eysturskarð et al. [25] also used live salmon lice, their sampling period was too short - only three days - to accurately estimate digestion time. In contrast, Staven et al. [17] conducted a comparable, longer-duration experiment, but with the use of frozen lice.

Our findings on the digestion time for salmon lice in lumpfish (6.4 and 12.9 days) differ significantly from those of Staven et al. [17], who estimated a digestion time of 29 hours at 9°C. While our results may initially seem lengthy, the observation of 12 salmon lice in a lumpfish stomach on day four of the experiment supports our conclusions. Since at least 11 of the lice were ingested *in situ* and the lumpfish had been at the research facility for at least seven days prior to being fed salmon lice, this suggests that the sedation and feeding procedure had minimal impact on digestion.

As the results from Staven et al. [17] were published while our experiments were still ongoing and it was already apparent that our results deviated substantially, we decided to conduct an additional experiment using frozen lice. However, no significant difference in digestion time was observed between frozen and live lice. This result aligns with the findings of Jackson et al. [28], who observed no significant differences in the digestibility of anchovy in Great Cormorants (*Phalacrocorax carbo* Linnaeus) based on whether the anchovy had been frozen prior to feeding. In addition to the freshness of the lice, other differences exist between our study and that of Staven et al. [17], including the feeding regime, lumpfish origin, and lumpfish size. However, none of these factors seem sufficient to fully explain the observed differences in digestion rates. Further research is needed to clarify these discrepancies.

In 1990, Hopkins and Larson investigated gastric evacuation of various food types in black and yellow rockfish (*Sebastes chrysomelas*), finding digestion times to vary based on food type. When fed fish, the rockfish displayed an intrinsic digestion pattern characterized by rapid evacuation initially, followed by a slower decline, typical for small, friable foods with low energy content. Conversely, when the rockfish ingested shrimps or crabs, the digestion pattern followed a linear decrease over time but included an initial lag phase [29]. Hopkins and Larson attributed the lag phase to the hydration of chitin, an enzymatic reaction requiring acidic conditions to initiate digestion of chitinous prey. They suggested that the secretion of chitinase and gastric acid, as well as the time needed for the exoskeleton to soften sufficiently for gastric musculature to break it down, accounted for the observed lag phase [29].

The digestion patterns observed in this study are like those reported when rockfish were fed shrimps or crabs, exhibiting an initial lag phase (Fig 3). This suggests that salmon lice are not easily digestible for lumpfish. Furthermore, the lag phase and overall digestion of adult female salmon lice were significantly longer than those of large mobile salmon lice (Fig 3). This difference is likely due to the larger size of adult females compared to adult males and preadult II salmon lice, indicating that food size plays a crucial role in determining digestion time in lumpfish. This observation aligns with the findings of Jobling [30], who proposed that differences in surface-to-volume ratios between larger and smaller food particles influence the rate of gastric emptying.

The significant difference in digestion times for adult female and large mobile salmon lice suggests that lumpfish might forage on a larger proportion of salmon lice at lower developmental stages than previously estimated based on stomach content analysis. In the Faroe Islands, the adult female to mobile salmon louse ratio in lumpfish stomachs sampled from salmon pens is approximately 1:1.2 (unpublished data). However, when accounting for the difference in digestion time, this ratio adjusts to approximately 1:2.1, which aligns more closely with the adult female to mobile salmon louse ratio on the salmon, typically varying between 1:2 and 1:4.2 [31]. This finding indicates that lumpfish might not be as selective in their foraging on adult female salmon lice as originally suggested [11–12].

Temperature is a critical factor influencing digestion time in fish by affecting metabolic rate, enzymatic activity, and gut motility. Numerous studies have demonstrated that water temperature significantly impacts digestion rates in fish [32–36]. In the current study, no significant differences were observed in the digestion time or degradation rate of salmon lice in lumpfish between temperatures of 6°C and 9°C. This may be due to the relatively small temperature range tested, as most studies investigating temperature effects typically examine larger temperature gaps. The main effect of temperature on degradation levels suggests faster degradation early in digestion or a higher initial degradation level at lower temperatures (Fig 2). This contrasts with the established understanding that higher temperatures typically accelerate fish digestion [32–36]. The authors find no biological explanation for this anomaly, attributing it to either random variation or an unrecorded experimental factor.

Physical activity has also been shown to influence digestion, with movement facilitating the circulation of digestive enzymes [28]. Conversely, insufficient physical activity can slow digestion, as highlighted by Ji and Li [37]. The lumpfish in present study were confined to 125-liter tanks and exhibited very limited physical activity outside feeding periods. This lack of movement may have contributed to reduced digestion efficiency and potentially masked the temperature-dependent differences in digestion rates typically observed. Future studies should therefore explore digestion rates over a broader range of temperatures and under varying levels of physical activity to better understand the interplay between these factors and digestion.

Additionally, because the salmon lice were fed to lumpfish in batches without individual tagging, it was not possible to track the developmental stage of the lice consumed by each fish. Given that the developmental stage of the lice significantly influences digestion time, this limitation hindered the ability to analyse the effects of other variables, such as lumpfish sex and size, on digestion rates. Future studies should thus aim to address these limitations by including detailed tracking of the developmental stages of prey and examining how additional factors influence digestion.

## Conclusions

To assess cleaning efficacy based on the number of lice found in lumpfish stomachs, it is crucial to understand the digestion time of salmon lice in lumpfish. In this study, the digestion time for large mobile lice was estimated to be 6.4 days, while for adult female lice, it was 12.9 days, at temperatures ranging from 6°C to 9°C. These results differ substantially from previously published findings [17]. While Staven et al. [17] suggested that the estimated number of salmon lice consumed per lumpfish per day could be calculated by dividing the number of lice found in a lumpfish stomach by 1.39, our results indicate a significantly lower cleaning efficacy. According to our findings, the number of retrieved salmon lice should be divided by 6.4 for large mobile lice and 12.9 for adult female lice to estimate the number of salmon lice consumed per lumpfish per day.

## Supporting information

S1 Logistic regression. The probability of recovering adult female and mobile salmon lice from lumpfish stomachs across different sampling times. Scatter points indicate proportion of lice recovered at each sampling point. Lines are based on logistic regression analysis of the data.
(TIF)

S2 Data. (XLSX)
