# Supplementary figures and images for: The digestion time for salmon louse (Lepeoptheirus salmonis) in lumpfish (Cyclopterus lumpus) in relation to freshness, developmental stage, and temperature
Source: PLoS One. 2025 Mar 13;20(3):e0311073. doi: 10.1371/journal.pone.0311073 (PMC11906037; doi:10.1371/journal.pone.0311073)

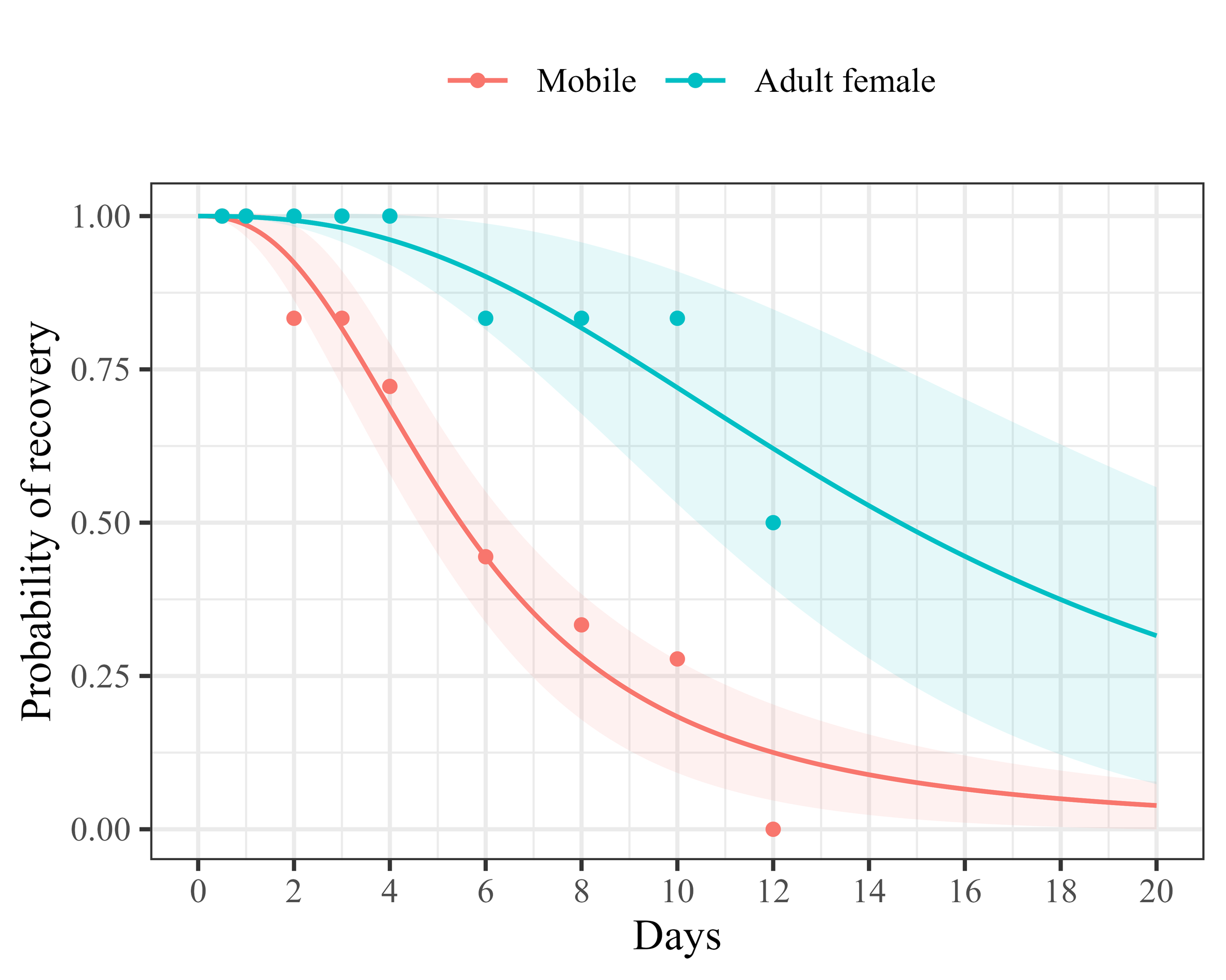

Supplement: S1 — The probability of recovering adult female and mobile salmon lice from lumpfish stomachs across different sampling times. Scatter points indicate proportion of lice recovered at each sampling point. Lines are based on logistic regression analysis of the data. (TIF) [file pone.0311073.s001.tif]
